# Feasibility of needle and syringe programs in Tajikistan distributing low dead space needles

**DOI:** 10.1186/s12954-018-0249-3

**Published:** 2018-08-31

**Authors:** William A. Zule, Alisher Latypov, David Otiashvili, Steffani Bangel, Georgiy V. Bobashev

**Affiliations:** 1 RTI International, 3040 E. Cornwallis Road, PO Box 12194, Research Triangle Park, NC 2709-2194 USA; 2 Global Health Research Center of Central Asia, Columbia University, New York, NY USA; 3 Addiction Research Center - Alternative Georgia, 14A Nutsubidze Street, Office 2, 0177 Tbilisi, Georgia; 4 Institute of Women and Ethnic Studies, 365 Canal St #1550, New Orleans, LA 70130 USA

**Keywords:** People who inject drugs, Central Asia, Heroin, Implementation science, Syringe exchange, Needle exchange

## Abstract

**Background:**

In 2012, the World Health Organization recommended that needle and syringe programs offer their clients low dead space insulin syringes with permanently attached needles. However, in many countries, these syringes are not acceptable to a majority of people who inject drugs. This study assessed the feasibility of working with needle and syringe programs to implement the WHO recommendation using low dead space detachable needles. The study also assessed the acceptability of the needles.

**Methods:**

Two needle and syringe programs in Tajikistan—one in Kulob and one in Khudjand—received 25,000 low dead space detachable needles each. The programs distributed low dead space detachable needles and a marketing flyer that emphasized the relative advantages of the needles. Each program also enrolled 100 participants, and each participant completed a baseline interview and a 2-month follow-up interview.

**Results:**

At follow-up, 100% of participants reported trying the low dead space detachable needles, and 96% reported that they liked using the needles. Both needle and syringe programs distributed all their needles within the first 60 days of the project indicating use of the needles, even among clients who did not participate in the study.

**Conclusions:**

This project demonstrates that it is feasible for needle and syringe programs to offer and promote low dead space needles to their clients. The findings indicate that low dead space needles are acceptable to needle and syringe program clients in these Tajikistan cities. To reduce HIV and hepatitis C virus transmission, needle and syringe programs should offer low dead space needles, low dead space insulin syringes in addition to standard needles, and syringes to their clients.

## Background

Needle and syringe programs (NSPs) reduce the spread of HIV and hepatitis C virus (HCV) among people who inject drugs (PWID). However, inadequate funding [1] and paraphernalia laws that criminalize needle and syringe possession [2] diminish the effectiveness of NSPs in many settings. Because the Global Fund to Fight AIDS, Tuberculosis and Malaria [3] is shifting funding from middle-income countries where many PWID live, efforts to increase funding for NSPs in the near future face serious challenges [4]. Additionally, despite attempts to change paraphernalia laws, they remain widespread [5]. Efforts to sustain and increase funding for NSPs and to advocate for the repeal of paraphernalia laws must be redoubled. However, success in these endeavors will take time. Meanwhile, PWID continue to share needles and syringes, and HIV and HCV continue to spread. Consequently, stopgap measures to slow HIV and HCV transmission remain important in the interim.

Low dead space (LDS) needles and syringes represent one such measure. The World Health Organization (WHO) reviewed evidence on the differential risks of HIV and HCV transmission associated with sharing 1-mL LDS insulin syringes with permanently attached needles compared with sharing high dead space (HDS) needles and syringes [6–9]. Following the review, the WHO concluded that the risks of HIV and HCV transmission associated with sharing LDS insulin syringes are lower than the risks associated with sharing HDS needles and syringes. In 2012, on the basis of this conclusion, the WHO recommended that NSPs offer their clients LDS insulin syringes [10, 11].

As part of the review process, the WHO commissioned a qualitative study to assess PWID’s preferences regarding LDS syringes. Participants in the qualitative study reported that it was important for syringes to come in different volumes and to have detachable needles [12]. To date, implementation of the WHO recommendation remains limited in regions where PWID use syringes with barrel capacities greater than 1 mL and where PWID strongly prefer detachable needles.

**Fig. 1:**
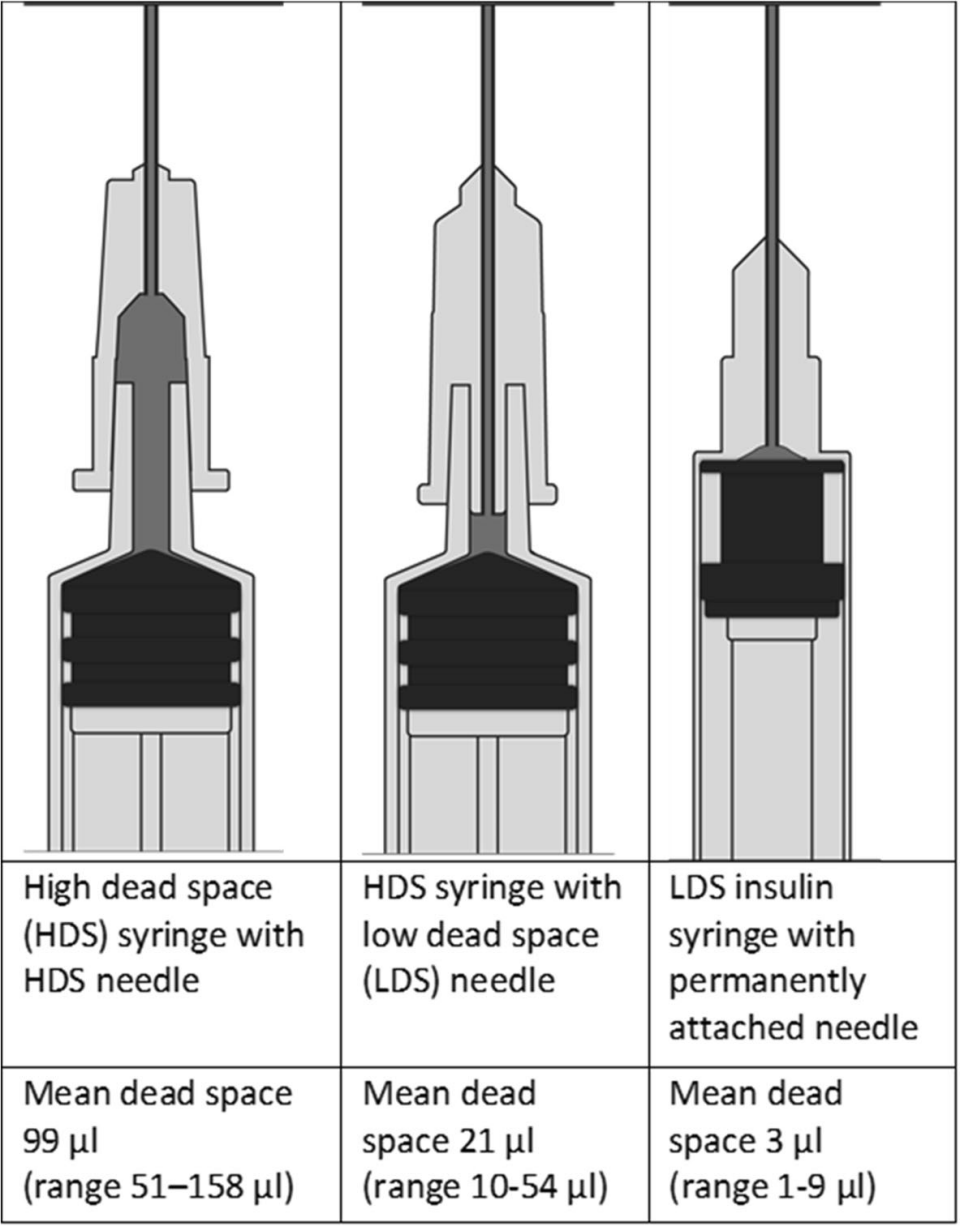
Illustrations of dead space in different needle and syringe combinations

A major harm reduction supplier, Exchange Supplies (www.exchangesupplies.org), developed LDS detachable needles that fit on standard HDS syringes of different volumes, thereby addressing the need for larger syringe barrels and preferences for detachable needles. These needles have the potential to significantly reduce the volume of dead space in a needle and syringe combination compared with standard detachable needles [13]. Figure 1 illustrates the dead space in an HDS needle attached to an HDS syringe (1.a), an LDS needle attached to an HDS syringe (1.b), and an LDS insulin syringe with a permanently attached needle (1.c). A recent laboratory study compared the relative effects of LDS insulin syringes, LDS detachable needles attached to HDS syringes, and HDS needle and syringe combinations on the recovery of HCV. The researchers recovered HCV from 98% of HDS needle and syringe combinations, 65% of LDS detachable needles attached to HDS syringes, and 47% of LDS insulin syringes immediately after use [14]; a study that compared the relative effects of LDS detachable needles and LDS insulin syringes on HIV survival reached similar conclusions [15].

Before implementing the WHO recommendation, NSPs must determine the lowest dead space option (i.e., LDS insulin syringes or LDS detachable needles) that is acceptable to their clients. A previously published report concluded that LDS insulin syringes were not compatible with the needs and preferences of NSP clients in Tajikistan [16]. The primary objectives of this study were to assess the feasibility of NSPs distributing LDS detachable needles and the acceptability of these needles to their clients. The study also sought to understand any barriers to using the needles and attributes of the needles that could affect uptake. Because NSPs in Tajikistan are very busy and have limited resources, distribution of the needles could not require extensive training or place an additional burden of NSP staff. Therefore, the research team developed a one-page flyer that described the benefits of LDS detachable needles for NSP staff to distribute.

## Methods

### Study design

#### Setting

The Republic of Tajikistan, population 8 million, shares a 1344-km border with Afghanistan. Poor socioeconomic conditions and its location on the main heroin trafficking route from Afghanistan contribute to cheap heroin, injection drug use, and HIV in Tajikistan [17]. PWID account for about half of all HIV infections in Tajikistan [17]. In 2015, 51 harm reduction sites throughout the country provided HIV prevention services to 13,456 PWID [18].

This project was conducted in two Tajikistan cities, Kulob and Khudjand. Kulob, with an estimated population of 100,000, is 120 miles southeast of the capital, Dushanbe. Khudjand, located 190 miles north of Dushanbe by car, has an estimated population of 170,000. Both cities are located on the Afghan opiate drug trafficking routes and have experienced significant increases in opiate consumption and in HIV and HCV infections among PWID over the past two decades [17]. The NSP in Kulob distributes approximately 52,500 needles/syringes to 750 PWID per month, whereas the NSP in Khudjand distributes 25,000 needles/syringes to 400 PWID per month.

Prior to the implementation of this study, the two NSPs distributed HDS detachable needles and syringes, but neither NSP distributes LDS detachable needles. Most of the syringes that they distributed were 2 mL, but they also distributed small numbers of 1-mL and 5-mL syringes. The HDS detachable needles ranged in length from 1 to 1.5 in. and ranged from 25 to 21 gauge in diameter. Both NSPs also distributed a small number of the 1-mL LDS insulin syringes with permanently attached needles. Both NSP continued to distribute this entire range of needles throughout the study period.

#### Study design

The pilot test used a pre-post-test design to assess the acceptability of LDS detachable needles among NSP clients, the feasibility of NSPs implementing a low-intensity intervention (i.e., distributing a flyer) to promote LDS detachable needles, and uptake of the needles by their clients.

#### Study sample eligibility and recruitment procedures

Eligibility criteria for participation in an interview included a minimum age of 18 years, self-report injecting at least once a week, self-report obtaining syringes from the NSP at least twice in the past 30 days, and report planning to remain in the city for the next 3 months. Both NSPs operated out of fixed sites and employed outreach workers who distributed syringes in the community. Outreach workers informed their clients and other people who injected drugs that they encountered in the community about the study. Staff at each fixed-NSP site informed clients who visited the NSP about the study. A trained NSP staff member at each site screened each potential participant after he or she expressed interest in the study. The NSP staff member described the study in greater detail to potential participants who met the eligibility criteria. Staff obtained informed consent from those who met the eligibility criteria and interested in participating. After obtaining informed consent, the NSP staff member collected locator information from the participant prior to the beginning of the interview.

#### Assessment procedures

Trained NSP personnel administered face-to-face interviews at study intake and at 2-month follow-up. Members of the research team, who were fluent in English, Tajik, and Russian, translated the questionnaire into Tajik and Russian prior to programming the questionnaires as computer-assisted personal interviews (CAPI). The following questions provide examples of questionnaire items that were used in the analyses:

1. How many times did you inject drugs in the last 30 days?
2. How many times in the last 30 days have you injected drugs with a needle that *was not* a low-waste needle?
3. How many times did you inject heroin mixed with dimedrol in the last 30 days?
4. Why did you use a needle that was not a low-waste needle? (responses are mutually exclusive)
  - I prefer these needles.
  - I could not get/I did not have a low-waste needle.
  - The low-waste needles are the wrong size.
  - The low-waste needles are of poor quality.
  - Other reason.

All study participants underwent rapid HIV testing at study intake. HIV testing was performed at the National AIDS Center located in each city. Additionally, dried blood spot specimens for HIV viral load testing were collected from the participants who tested positive for HIV.

#### Intervention

Social cognitive theory and diffusion of innovations theory [18, 19] provided the theoretical basis for the intervention. According to the social cognitive theory, learning must be accompanied by facilitation (i.e., removal of barriers) before information can be acted on [20]. For this research, the study donated LDS detachable needles to the participating NSP in each city. Subsequently, NSP outreach workers distributed flyers that described the advantages of LDS detachable needles to their clients and began offering their clients LDS detachable needles.

**Fig. 2:**
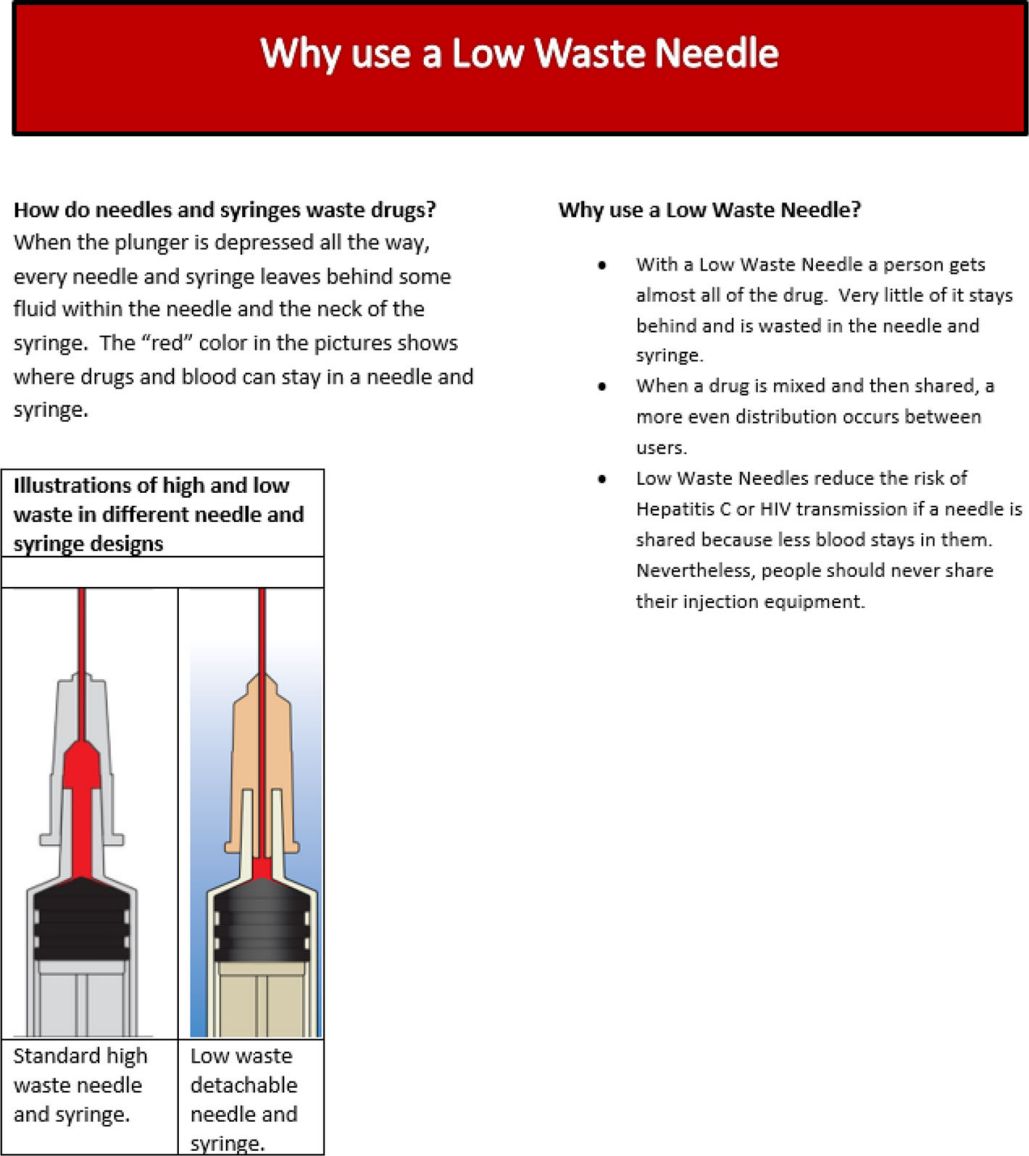
Image of marketing flyer to promote LDS detachable needles

The flyer referred to the needles as “low-waste” and emphasized the following relative advantages of LDS detachable needles (Fig. 2): (1) LDS detachable needles reduce the amount of drug that is retained and wasted in the dead space; (2) LDS detachable needles facilitate accurate division of liquefied drug solution between two PWID; and (3) LDS detachable needles reduce the volume of blood retained in a needle and syringe after use, which may decrease the risk of HIV and HCV transmission if they are shared. Previous research suggested that these advantages would be compatible with the values and beliefs of PWID in Tajikistan [16].

The research team explained the purpose study to the outreach workers and answered any questions that they had regarding the flyers and the LDS detachable needles. Outreach workers did not receive any additional training on what to tell people when they distributed the flyers.

Each participant received 20 LDS detachable needles at enrolment. Participants could obtain additional LDS detachable needles from the NSP fixed site or outreach workers until all the needles were distributed.

### Measures

#### Feasibility

The primary feasibility measures included NSP distribution of LDS detachable needles and NSP distribution of flyers.

#### Penetration

In addition to the 100 NSP clients in each city who participated in the study, each NSP offered the LDS detachable needles to all their clients. The time required for an NSP to distribute all 25,000 needles received provides an indirect measure of penetration of the intervention (i.e., LDS detachable needles) into the population of NSP clients.

#### Acceptability

The follow-up questionnaire included two questions to assess the acceptability of the LDS detachable needles. The first question asked if a participant had used LDS detachable needles; the second asked if the participant liked using them.

#### Attributes

To assess the attributes of LDS detachable needles that may affect adoption, participants who responded that they liked using LDS detachable needles were asked additional questions regarding relative advantages, such as reduced wasted drug, increased accuracy of dividing liquefied drug solution, and less retention of blood after use. Participants were also asked if they observed other PWID injecting with an LDS needle and if they told other PWID about using LDS detachable needles.

#### Uptake

Measures of uptake included (1) any use of LDS detachable needles in the past 30 days, (2) no use of LDS detachable needles in the past 30 days, and (3) and number of injections with an HDS needle in the past 30 days. To assess the factors that affected the uptake, participants who reported using any HDS needles in the past 30 days were asked why they used the HDS needles.

#### Sustainability

The United Nations Development Programme (UNDP), the principal recipient of the Global Fund grants in Tajikistan, procures needles and syringes and distributes them to NSPs across the country. This arrangement prevented the participating NSPs from purchasing LDS detachable needles on their own. Therefore, this pilot study did not assess sustainability.

### HIV antibody testing and HIV viral load testing

All HIV antibody testings in Tajikistan are performed at AIDS Centers that are run by the government of Tajikistan. Following completion of the baseline interview, an NSP staff member transported each participant to the local AIDS Center where a staff member trained in HIV testing and counseling performed rapid HIV antibody testing. If a participant tested positive for HIV antibodies, an AIDS Center staff member collected blood by finger stick onto filter paper as dried blood spots and stored them in freezers at − 20 °C. The dried blood spots were transported from Tajikistan to the University of North Carolina at Chapel Hill Virology Laboratory, which performed the HIV viral load testing.

### Analysis

The initial analyses used contingency tables and *t* tests to compare participants in the two cities. The NSP in each city ran out of LDS detachable needles before most participants completed their follow-up interview. Therefore, our analyses focused on two alternative outcomes. The first analyses used logistic regression analysis to examine the factors associated with preferring HDS needles. The second analyses used negative binomial regression to examine the factors associated with the number of injections in the past 30 days with an HDS needle. Analyses of both outcomes were limited to Khudjand because only one participant in Kulob preferred HDS needles, and all but one person in Kulob reported the reason for using an HDS needle was because they were unable to obtain an LDS needle.

The study team initially identified potential independent variables for the model based on previous qualitative work and an examination of injecting practices in the two cities reported at baseline. Because both sites distributed all their LDS needles before they completed all their follow-up interviews, we also considered additional variables that were likely to affect access to LDS detachable needles, such as frequency of injection and the number of days from the start of the study until a participant completed his or her follow-up interview. For the logistic regression model, variables that were significant (*p* <  0.2) in bivariate analyses were entered into the multivariable model using a forward stepwise variable selection procedure with criteria for entry set at *p* <  0.20 and removal at *p* <  0.25. The multivariable negative binomial regression model initially included all variables that were significant at *p* <  0.20 in the bivariate analyses. Variables with the highest *p* values were removed manually from the model one at a time until only variables with *p* values < 0.20 remained.

### Ethical approval and informed consent

The study received ethical approval from the Committee of Medical Ethics of Tajikistan and the RTI International Office of Human Research Protection. All participants provided oral informed consent and received a food package valued at the equivalent of $5 USD for their participation in their initial interview and $10 USD for participation in their follow-up interview. A Data and Safety Monitoring Board also reviewed and approved all study procedures.

## Results

### Pilot test sample

Data collection for the study began in August 2015 and ended in November 2015. The sample included 200 participants (100 per city) who were recruited over a 1-month period in each city. Among the sample, three fourths were male, with a mean age of approximately 38 years. Study participants in Kulob and Khudjand differed in potentially important respects, as shown in Table 1. Almost 100% (199/200) of participants completed a 2-month follow-up interview.

**Table 1:** Characteristics of the sample (*n* = 199), by city—Kulob and Khudjand

	Kulob (*n* = 99)	Khudjand (*n* = 100)	*p* value
Background characteristics			
Mean age (standard deviation [SD])	35.9 (8.4)	39.3 (7.3)	
Percentage male	96.0	77.0	< 0.001
Percentage high school education or greater	53.0	81.0	< 0.001
Percentage unemployed	96.0	51.0	< 0.001
Percentage married or living as married	32.3	55.0	0.001
Percentage ever in substance abuse treatment	75.5	45.0	< 0.001
Percentage ever incarcerated 1 year or more	27.3	41.0	0.041
Percentage ever had a sexually transmitted infection	9.1	16.0	0.199
Percentage tested positive for HIV	22.2	31.0	0.161
Percentage hazardous or harmful drinking^a^	42.4	51.0	0.225
Percentage reported using heroin daily with or without dimedrol during the past 30 days	90	8	< 0.001
Drug use and injecting practices			
Mean number of injections past 30 days (SD)	67.3 (23.6)	21.9 (17.1)	< 0.001
Mean number of injections with a high dead space needle in past 30 days (SD)	48.2 (35.2)	11.2 (14)	< 0.001
Mean number of times injected heroin by itself in the past 30 days (SD)	15.3 (24.1)	1.5 (6.2)	< 0.001
Mean number of times injected heroin mixed with dimedrol in the past 30 days (SD)	52.0 (34.1)	20.3 (17.6)	< 0.001
Percentage any direct or indirect needle or syringe sharing in the past year	9.1	6.0	0.409
Mean number of days between project start and completion of follow-up interviews (SD)	81.8 (9.5)	78.7 (8.9)	0.019
Percentage injected homemade opiates in past 30 days	0.0	14.0	< 0.001
Percentage injected methamphetamine or another stimulant in past 30 days	0.0	0.0	–
Percentage injected prescription opiate in past 30 days	0.0	1.0	1.000
Percentage injected other prescription drug	0.0	11.0	0.001
Percentage injected any other drug	0.0	0.0	–
Do you like using low dead space needles?	99.0	92.9	0.065
What do you like about using low dead space needles?			
Percentage less wasted drugs	94.9	51.0	< 0.001
Percentage they are free	57.6	99.0	< 0.001
Percentage it is easy to split drugs accurately	86.9	18.0	< 0.001
Percentage they save me money on drugs	85.9	64.0	< 0.001
Percentage they are healthier for me to use because less infected blood stays in them	99.0	24.0	< 0.001
Percentage did not use any high dead space needles	21.2	31.0	0.117
Reasons for using high dead space needles in past 30 days at follow-up	(*n* = 78)	(*n* = 69)	< 0.001
Percentage prefer high dead space needles	1.3	44.9	< 0.001
Percentage could not get a low dead space needle	98.7	40.6	< 0.001
Percentage low dead space needles wrong size	0.0	13.0	0.001
Percentage low dead space needles poor quality	0.0	1.4	0.469

^a^Based on Alcohol Use Disorders Identification Test (AUDIT) classification score of 8 or higher (https://www.drugabuse.gov/sites/default/files/files/AUDIT.pdf)

### Feasibility and penetration

During the study, both NSP offered all clients their choice of HDS needles or LDS needles until they ran out of LDS needles. All LDS needles at each site were distributed in less than 2 months after the NSP began offering them. The NSP in Khudjand distributes approximately 25,000 needles/syringes per month, which means that LDS needles represented about 50% of all needles/syringes distributed during the study period at this NSP. The NSP in Kulob distributes 52,000 needles/syringes per month, which means that LDS needles represented about 25% of the needles/syringes distributed during the study period at this site.

### Acceptability

NSP staff members in each city reported that many of their clients preferred the LDS needles. At the 2-month follow-up interview, 100% of participants in the pilot test reported trying an LDS detachable needle, and 96% reported that they liked using the needles.

### Attributes of LDS detachable needles and rate of adoption

At follow-up, 100% of study participants reported seeing the flyer that explained the relative advantages of using LDS needles. The two most frequently mentioned advantages of LDS needles were less drug waste (73%) and less blood retention (61%) after use. Most (96%) study participants reported observing other PWID using an LDS needle, and 96% reported telling their friends about LDS needles. In Kulob, 95% of participants reported that they liked LDS needles because the needles wasted less of their drug, and 99% reported they liked that the needles retained less blood and may reduce their risk of HIV and HCV. In Khudjand, only 51% endorsed liking LDS needles because they wasted less of their drug and only 24% endorsed liking LDS needles because they retained less blood and may reduce their risk of infections.

### Uptake

From the beginning to end, the enrollment and follow-up process took 104 days in Khudjand and 91 days in Kulob; as noted earlier, both NSPs distributed all their LDS detachable needles within the first 60 days. Only one participant in Kulob reported using HDS needles because he preferred them, and 98 participants in Kulob reported using that they used an HDS needle at follow-up because they could not get a LDS detachable needle. We do not present models for Kulob because the distribution of these responses resulted in numerically unstable models for both outcomes.

#### Prefer HDS needles

In bivariate analyses in Khudjand, usually using 1-mL syringes was negatively associated with preferring HDS needles (odds ratio [OR] 0.36; 95% confidence interval [CI] 0.13, 1.01; *p* = 0.051). The number of injections of dimedrol mixed with heroin (OR = 1.03; 95% CI = 1.00, 1.05; *p* = 0.045) and the total number of injections in the past 30 days (OR = 1.04; 95% CI 1.04, 1.08; *p* = 0.015) were both positively associated with preferring HDS needles (see Table 2). In the multiple logistic regression model, usually using 1-mL syringes was negatively associated with preferring HDS needles (OR = 0.30; 95% CI = 0.10, 0.92), and the total number of injections in the past 30 days was positively associated with preferring HDS needles (OR = 1.04; 95% CI = 1.01, 1.07; *p* = 0.014).

**Table 2:** Logistic regression model for participants in Khudjand who prefer high dead space needles to low dead space needles

Variables	Bivariate		Multivariable	
	Odds ratio (95% CI)	*p* value	Odds ratio (95% CI)	*p* value
Gender (female = 0, male = 1)	1.36 (0.48, 3.88)	0.562		
Age in years	0.99 (0.93, 1.05)	0.715		
High school education or more	0.41 (0.15, 1.15)	0.092		
Pharmacy main source of syringes at follow-up	1.87 (0.72, 4.86)	0.198	2.12 (0.74, 6.11)	0.162
Usually used 12-mm/ 0.5-in. needles	0.58 (0.17, 1.94)	0.378		
Usually used 1-mL syringes	0.36 (0.13, 1.01)	0.051	0.30 (0.10, 0.92)	0.035
Injected homemade opiates at follow-up	0.56 (0.15, 2.19)	0.408		
Like LDS needles because they waste less drugs	1.04 (0.44, 2.42)	0.935		
Like LDS needles because they make it easier to split drugs accurately	0.58 (0.17, 1.94)	0.378		
Like LDS needles because they save money on drugs	0.69 (0.29, 1.65)	0.408		
Like LDS needle because they are healthier to use because less infected blood stays in them	0.89 (0.33, 2.44)	0.824		
Number of injections with heroin mixed with dimedrol in the past 30 days	1.03 (1, 1.05)	0.045		
Number of injections with heroin by itself in the past 30 days	1.07 (0.98, 1.17)	0.142		
Total number of injections past 30 days	1.04 (1.01, 1.08)	0.015	1.04 (1.01, 1.07)	0.014

#### Number of injections with HDS needles

In bivariate analyses using negative binomial regression, male gender (incidence rate ratio [IRR] = 2.05; 95% CI = 1.25, 3.37; *p* value = 0.005), preferring HDS needles (IRR = 1.95; 95% CI = 1.25, 3.02; *p* = 0.003), number of injections of dimedrol mixed with heroin (IRR = 1.04; 95% CI = 1.02, 1.05; *p* <  0.001), and the total number of injections (IRR = 1.06; 95% CI = 1.04, 1.08; *p* <  0.001) were all positively associated with the number of injections using an HDS needle (Table 3). Using an HDS needle because a participant was unable to get an LDS detachable needle was negatively associated with the number of injections with an HDS needle (IRR = 0.55; 95% CI 0.36, 0.83; *p* = 0.004),

**Table 3:** Negative binomial regression for frequency of using HDS needles in Khudjand

	IRR (95% CI)	*p* value	IRR (95% CI)	*p* value
Gender (female = 0, male = 1)	2.05 (1.25, 3.37)	0.005		
Age in years	0.98 (0.95, 1.01)	0.202		
Completed high school or more (no = 0, yes = 1)	0.72 (0.43, 1.21)	0.217		
Prefers regular needles to LDS needles = 1.00	1.95 (1.25, 3.02)	0.003		
Pharmacy main source of syringes	1.48 (0.92, 2.38)	0.106		
Unable to get LDS	0.55 (0.36, 0.83)	0.004	0.60 (0.39, 0.91)	0.016
Injected homemade opiates	1.48 (0.83, 2.66)	0.187	1.54 (0.85, 2.79)	0.158
Like LDS because they waste less drugs	0.94 (0.63, 1.42)	0.775		
Like LDS because needles because they are free	1.87 (0.22, 15.69)	0.564		
Like LDS needles because they make it easier to split drugs accurately	0.70 (0.41, 1.19)	0.187		
Like LDS needles because less infected blood stays in them	0.81 (0.50, 1.31)	0.397		
Usually used 12-mm/ 0.5-in. needles	0.91 (0.53, 1.55)	0.730		
Usually used 1-mL syringes	1.02 (0.66, 1.58)	0.932		
Number of times injected heroin mixed with dimedrol in the last 30 days?	1.04 (1.02, 1.05)	< 0.001		
Number of times injected heroin by itself in the last 30 days?	1.02 (0.99, 1.05)	0.166		
Number of injections in the last 30 days?	1.06 (1.04, 1.08)	< 0.001	1.06 (1.04, 1.08)	< 0.001
Number of days between the start of the study and follow-up interview completed	1.00 (0.98, 1.02)	0.931		

In the multivariable model, being unable to get an LDS detachable needle (IRR = 0.60; 95% CI = 0.39, 0.91; *p* = 0.016) was negatively associated with the number of injections with an HDS needle (IRR = 1.06; 95% CI = 1.04, 1.08; *p* <  0.001).

### Impact of LDS detachable needles on HIV viral burden

Dried blood spots from 48 of the 54 participants who tested positive for HIV were tested successfully for HIV viral load. Six samples were damaged during collection, storage, or transport. HIV viral load ranged from undetectable (< 50 copies/mL) to 1,280,192 copies/mL. The mean number of copies was 128,323 (SD 346,521) and median 1952 copies/mL. LDS needles attached to HDS syringes—assuming 21 μL of dead space—would retain approximately 0.029 μL of blood after injection and rinsing [13]. At the mean HIV viral load, 0.029 μL of blood would contain approximately 4 copies of HIV; at the median, it would contain less than 1 copy of HIV. At the mean viral load, the 1 μL of blood retained in an HDS needle attached to an HDS syringe after injection and rinsing [21] would contain 128 copies of HIV; at the median, it would contain about 2 copies of HIV. In contrast, the 0.001 μL of blood retained in an LDS insulin syringe with a permanently attached needle after injection and rinsing [21] would contain less than 1 copy of HIV at both the median and mean HIV viral loads.

## Discussion

At follow-up, 100% of participants reported trying the LDS detachable needles. This followed a simple intervention (i.e., distributing LDS detachable needles and a flyer that explained their benefits). Both NSPs in the study distributed the flyer and needles without difficulty. According to NSP records, both NSPs distributed all (25,000 per NSP) of their LDS detachable needles in less than 2 months. This suggests that the LDS detachable needles achieved high penetration among NSP clients beyond the study sample. Although other reports have examined the acceptability of LDS detachable needles and syringes among NSP clients [16, 22], this is one of the first studies to demonstrate the feasibility of NSPs influencing their clients to use LDS detachable needles.

The flyer seemed to be particularly effective at marketing the LDS needles to participants in Kulob where 95% endorsed liking LDS needles because they waste less of their drug, 87% endorsed liking them because they made it easier to split drugs accurately, and 99% endorsed liking them because they retained less blood and may reduce their risk of HIV and HCV. Moreover, almost all use of HDS needles at follow-up seemed to be because participants were unable to obtain LDS detachable needles. In contrast, the flyers seemed to be much less effective at marketing LDS detachable needles in Khudjand. Only 51% of participants endorsed liking LDS because they wasted less drug, 18% endorsed liking them because they made it easier to split drugs accurately, and 24% endorsed liking them because they retained less blood and may reduce risk of HIV and HCV. Nevertheless, even in Khudjand, 31% reported that they did not use any HDS needles in the past 30 days at follow-up, and only 45% (*n* = 31) of those who did use an HDS needle reported that they preferred HDS needles. Moreover, preferring HDS needles was not significant in the multivariable model for the number of injections using an HDS needle.

### Potential impact of switching to LDS detachable needles

Studies point to a lower risk of HIV and HCV transmission associated with LDS needles attached to HDS syringes compared with HDS needle and syringe combinations [14, 15]. These studies tested the first-generation LDS detachable needles. When first-generation 25 g LDS detachable needles were attached to a 2-mL HDS syringe, the dead space was 54 μL, whereas the dead space in the current (third-generation) generation of 25 g LDS detachable needles attached to a 2-mL HDS syringe is 17 μL (A. Preston, Exchange Supplies, personal communication, November 17, 2016). Consequently, it is likely that the reduction in risk associated with the current generation of LDS detachable needles is greater than the reduction observed in those studies [13].

### Limitations

This study did not include a comparison arm, which weakens our ability to attribute changes to the project [23]. However, prior to the study, LDS detachable needles were not available in either of the study cities. During the study, there were no other potential sources of LDS detachable needles, and no other organizations were encouraging PWID to use LDS detachable needles. Therefore, attributing the use of LDS detachable needles to the intervention is reasonable.

Both NSP ran out of LDS detachable needles while the data collection was in progress, which made it impossible for the participants to continue using the needles. Therefore, we cannot be certain how many participants would have continued using the needles if they had been available. However, at follow-up, 71% of people who reported any injections in the past 30 days with other types of needles reported that they did so because they were unable to obtain an LDS detachable needle. Another limitation is that this study did not test strategies for NSP to switch their clients from HDS needles to LDS insulin syringes with permanently attached needles, which retain substantially less blood after use than the LDS detachable needles retain. Natural experiments have shown that under certain conditions, people who inject drugs will switch from HDS detachable needles to LDS insulin syringes with permanently attached needles [24]. However, it is not clear if similar changes would occur in settings where people inject volumes of fluid greater than 1 mL and the largest LDS insulin syringes with permanently attached needles are 1 mL.

## Conclusions

The pilot study findings have important implications for NSPs that are considering offering LDS equipment. In areas where most PWID will not use LDS insulin syringes, NSPs should offer LDS detachable needles in addition to LDS insulin syringes. More generally, the study findings highlight the importance of balancing the selection of interventions for implementation based on a combination of efficacy and acceptability. Highly efficacious interventions that are not compatible with the values and beliefs of both the intended recipients and the service providers who will be delivering them may have limited impact. While striving to implement perfect solutions to problems, policymakers, harm reductionists, and public health workers must not let the quest for perfection prevent or delay implementation of viable and feasible solutions, such as distributing LDS detachable needles.

## Abbreviations

CAPI: Computer-assisted personal interview
HCV: Hepatitis C virus
HDS: High dead space
HIV: Human immunodeficiency virus
LDS: Low dead space
NSP: Needle and syringe program
PWID: People who inject drugs
SD: Standard deviation
UNDP: United Nations Development Program
USD: United States Dollar
WHO: World Health Organization
